# Lessons learnt and challenges in adopting the ECDC and who Ebola guidelines at Mater Dei Hospital

**DOI:** 10.1186/2047-2994-4-S1-P5

**Published:** 2015-06-16

**Authors:** N Abela, E Tartari Bonnici, AR Parascandalo, MA Borg

**Affiliations:** 1 Infection Control Unit, Mater Dei Hospital, Siggiewi, Malta; 2 Infection Control Unit, Mater Dei Hospital, B\Kara, Malta; 3 Infection Control Unit, Mater Dei Hospital, Naxxar, Malta; 4 Infection Control Unit, Mater Dei Hospital, Mosta, Malta

## Introduction

Ebola is one of the Viral Haemorrhagic Fever (VHF) infections. The EVD outbreak in West Africa represents a major public health issue. EVD is a serious occupational health and safety risk for healthcare workers (HCWs). Mater Dei Hospital, the main tertiary care hospital in Malta organised and prepared for a potential confirmed or suspected case.

## Objectives

1. Reviewing international EVD guidelines

2. Developing a framework for protection of HCWs

3. Procurement of PPE’s

4. Minimise the risk of transmission to HCWs coming in contact with suspected or confirmed cases

## Methods

During the preparedness for the admission of a potential EVD case, the infection control unit in the tertiary care hospital in Malta guided the selection process of different types of PPE supplies according the WHO and ECDC guidelines. Resource management including stock management, availability of different sizes and shapes of PPE was carried out. Written protocols including technical specifications for medical devices and PPEs needed to be in place.

## Results

Various members of the core group for the managemet of potential EVD positive patients failed their N95 respirator fit test. Heat-associated stress and fogging of the goggles with severe impaired visibility were two particular concerns from healthcare workers necessitating the abrupt interruption of practice due to unbearable working conditions. Responses to prolonged training and probation of supplies identified the best preferred option to be the use of PAPR rather than goggles and particulate respirator (N95), the former providing comfort and a sense of protection.

## Conclusion

A fundamental principle guiding the selection of PPE for the management of EVD affected patients has to take in consideration ease of use, dexterity, comfort, minimal levels of heat-associated stress and climate conditions for healthcare workers spending a considerable amount of time inside a patient's room.

## Disclosure of interest

None declared.

